# Roles of the Endogenous Lunapark Protein during Flavivirus Replication

**DOI:** 10.3390/v13071198

**Published:** 2021-06-22

**Authors:** Pham-Tue-Hung Tran, Naveed Asghar, Magnus Johansson, Wessam Melik

**Affiliations:** Inflammatory Response and Infection Susceptibility Centre (iRiSC), School of Medical Science, Örebro University, 703 62 Örebro, Sweden; hung.tran@oru.se (P.-T.-H.T.); naveed.asghar@oru.se (N.A.)

**Keywords:** flavivirus, Kunjin virus (WNV_KUN_), Langat virus (LGTV), Zika virus (ZIKV), replication, replicon-expressing cell line, lunapark (LNP), NS4B

## Abstract

The endoplasmic reticulum (ER) of eukaryotic cells is a dynamic organelle, which undergoes continuous remodeling. At the three-way tubular junctions of the ER, the lunapark (LNP) protein acts as a membrane remodeling factor to stabilize these highly curved membrane junctions. In addition, during flavivirus infection, the ER membrane is invaginated to form vesicles (Ve) for virus replication. Thus, LNP may have roles in the generation or maintenance of the Ve during flavivirus infection. In this study, our aim was to characterize the functions of LNP during flavivirus infection and investigate the underlying mechanisms of these functions. To specifically study virus replication, we generated cell lines expressing replicons of West Nile virus (Kunjin strain) or Langat virus. By using these replicon platforms and electron microscopy, we showed that depletion of LNP resulted in reduced virus replication, which is due to its role in the generation of the Ve. By using biochemical assays and high-resolution microscopy, we found that LNP is recruited to the Ve and the protein interacts with the nonstructural protein (NS) 4B. Therefore, these data shed new light on the interactions between flavivirus and host factors during viral replication.

## 1. Introduction

The genus *Flavivirus* (family *Flaviviridae*) consists of important zoonotic viruses causing morbidity and mortality worldwide. Within the genus, the mosquito-borne neurotropic West Nile virus (WNV) may cause severe meningoencephalitis [1]. Similarly, Zika virus (ZIKV) is another mosquito-borne flavivirus, which has gained public attention by causing congenital microcephaly or Guillain–Barré syndrome at recent outbreaks in 2014–2015 [2]. In addition, there are also tick-borne flaviviruses causing neurotropic diseases. Among them, infection with tick-borne encephalitis virus (TBEV) Far-Eastern subtype may result in severe encephalitis with mortality rates as high as 20–30% in Eurasia [3,4].

Flaviviruses are enveloped, single-stranded positive-sense RNA viruses with an icosahedral structure. The viral genome is about 11 kb with one open reading frame (ORF) encoding a polyprotein. Cleavages of the polyprotein by host and viral proteases at the endoplasmic reticulum (ER) membrane result in three structural proteins: capsid (C), precursor membrane (prM), envelope (E); and seven non-structural proteins (NS; NS1 to NS5). The viral genome also contains two untranslated regions (UTRs) flanking the ORF [5].

During flavivirus infection, the ER membrane invaginates into the luminal side, forming packets of bilayer membrane vesicles (Ve), where the replication complex (RC) of NS proteins and the viral RNA are located [5,6,7]. The viruses replicate their RNA genome at the generated Ve, followed by genome encapsidation and envelopment. New particles transit from the ER to the Golgi, followed by a particle maturation process at the trans-Golgi network. Ultimately, the newly formed viruses are released from infected cells by exocytosis.

At the Ve, NS2A, NS2B, NS4A, and NS4B are transmembrane (TM) proteins, acting as scaffolds for the RC. Overexpression of NS4A or NS4B can trigger the ER membrane invagination, which results in vesicles morphologically similar to the Ve [8,9,10]. Before cleavage of the polyprotein, NS4A and NS4B are bridged by a conserved 23-amino-acid-long signal peptide with a molecular weight of 2,000 Da (2K). The two proteins are cleaved by the viral protease NS2B/NS3 and a host peptidase [8].

In eukaryotic cells, the ER is a large membrane-based organelle that spreads throughout the cytoplasm presenting three major morphologies: the nuclear envelope, the peripheral ER cisternae, and the interconnected tubular network [11]. To induce and maintain the highly curved tubular structure, host machinery is employed, including the reticulon (RTN) family of integral membrane proteins, the DP1/Yop1 proteins [12,13], the atlastin (ATL) family of dynamin-like GTPases [14,15], and the lunapark (LNP) protein [16,17]. Due to their membrane-remodeling capacities and the location within ER, these host proteins may have roles in flavivirus replication and the formation of the Ve. It has previously been shown that silencing of RTN3.1A [18], ATL2, or ATL3 [19,20] resulted in reductions of flavivirus titers. However, the putative roles of the other ER membrane-shaping proteins, such as LNP, remains to be clarified during flavivirus infection.

In this study, we aimed to characterize the functions of the host LNP during flavivirus infection. We used the Kunjin strain of WNV (WNV_KUN)_ and ZIKV strain SL1602 Suriname 2016, as mosquito-borne flavivirus models. In addition, we used Langat virus strain TP21 (LGTV), which belongs to the TBEV serocomplex [3,21], as the tick-borne flavivirus model. We generated cell lines expressing the WNV_KUN_ and LGTV replicons to study the role of LNP during virus replication. We found that the LNP protein is essential for virus replication and maintenance of the Ve. Conclusively, we demonstrate that the flavivirus NS4B recruits LNP to the Ve at its C-terminus, which suggests a role for LNP within the underlying mechanism of Ve generation.

## 2. Materials and Methods

### 2.1. Cell Culture

Baby hamster kidney (BHK-21), Vero B4, human embryonic kidney 293 (HEK293), and A549 cells were maintained in Dulbecco’s Modified Eagle’s Medium (DMEM) containing 1 g/L glucose (Gibco, Paisley, UK) supplemented with 10% heat-inactivated fetal bovine serum (HI‒FBS) (Gibco), 100 U/mL penicillin–streptomycin (PEST) (Gibco), and 1% nonessential amino acids (NEAA) (Gibco) at 37 °C in 5% CO_2_.

### 2.2. Viruses

An infectious clone of WNV_KUN_ based on FLSDX sequence (accession number AY274504) was rescued. In short, the sequences were chemically synthesized and cloned into DNA vectors to generate two constructs: pKUNV-CME and pKUNV-luc-rep. pKUNV-CME comprises an SP6 promoter sequence and the 5′NCR, the structural genes, and 668 bp of NS1 of WNV_KUN_, whereas pKUNV-luc-rep is an established KUNV replicon where most of the structural cassette is replaced with firefly luciferase reporter gene as described previously [3]. The plasmids were cleaved by suitable restriction enzymes and ligated to generate a pSP6-KUNV construct with an SP6 promoter upstream the 5′-UTR and the hepatitis delta virus ribozyme (HDVr) sequence immediately downstream of the 3′-UTR of the complete KUNV sequence. The cloning work was performed using standard molecular biology procedures and verified by DNA sequencing (Eurofins MWG Operon, Germany). The pSP6-KUNV construct was linearized prior to RNA synthesis followed by capping to generate a complete WNV_KUN_ RNA genome, using MEGAscript SP6 in vitro transcription kit (Ambion, USA) as per the manufacturer’s instructions. The capped RNA-transcripts were transfected into a mixed (1:1) population of HEK293 and Vero B4 cells to rescue the infectious virus as described previously [22]. LGTV strain TP21 was a kind gift from A. K. Överby (Department of Clinical Microbiology, Virology, Umea University, Sweden). ZIKV strain SL1602 (accession number KY348640) originated from Suriname was obtained from the European Virus Archive.

### 2.3. Preparation of Gene Constructs

The WNV_KUN_ replicon was constructed based on the WNV_KUN_ sequence (accession number AY274504), as described previously [3]. Briefly, the replicon is driven by a T7 promoter expressing an ORF of WNV_KUN_ where most of the structural cassette is replaced with firefly luciferase reporter gene (*Luc*). The foot-and-mouth disease virus autoprotease 2a sequence was fused in frame after *Luc* for post-translational cleavage release of the reporter. The ORF is flanked by the 5′-UTR and the 3′-UTR and a hepatitis delta virus antigenomic ribozyme sequence is inserted immediately downstream of the WNV_KUN_ 3′-UTR followed by the Simian virus 40 polyadenylation signal. An IRES sequence and a NeoR/KanR gene which confers G418 antibiotic resistance was inserted into the 3′-UTR sequence. The LGTV replicon was constructed similarly, and its sequence is based on the LGTV strain TP21 (accession number NC003690).

The TBEV Torö-2003 strain (accession number AH013799) was used as a template to clone NS4A-NS4B, NS4A, 2K-NS4B, 2K-NS4B TM1‒3, 2K-NS4B TM1, anchor-prM-E genes into the plasmid pmCherry (Clonetech) introducing a C-terminal mCherry tag to the proteins.

pHAGE2 Lnp-mCherry was a gift from Tom Rapoport (Addgene plasmid # 86687; http://n2t.net/addgene:86687; accessed on 18 June 2021; RRID:Addgene_86687) [23].

### 2.4. Establishment of Cell Line Expressing RNA WNV_KUN_ or LGTV Replicons

The WNV_KUN_ or LGTV DNA-based replicon construct was linearized by ClaI enzyme, followed by in vitro transcription and cap analog incorporation using mMESSAGE mMACHINE™ T7 Transcription Kit (Invitrogen, Vilnius, Lithuania). The RNA was purified using RNeasy Mini Kit (Qiagen, Hilden, Germany) prior to transfection into BHK-21 cells by Lipofectamine™ MessengerMAX™ Transfection Reagent (Invitrogen). Two days after transfection, the cell culture was supplemented with 600 µg/mL G418 (Invivogen, Toulouse, France) to select transfected cells.

### 2.5. Transfection

70% confluent A549 cells in a 24-well plate were incubated with a premix of 100 µL OptiMEM medium (Gibco), 10 pmol siRNAs against LNP (Catalogue number: L-023148-01-0005, Horizon Discovery, Cambridge, UK) or non-targeting (NT) siRNA (Catalogue number: D-001810-01-20, Horizon Discovery, UK), and 1 µL of lipofectamine RNAiMAX reagent (Invitrogen) for 24 h, according to the manufacturer’s instructions. For BHK-21 replicon stable cell lines, the treatment was repeated twice.

50–100 ng of the LNP-mCherry construct was transfected in A549 cells grown in 24 well-plates, using Lipofectamine 2000 (Invitrogen).

### 2.6. Virus Infections and Cell Harvesting

After transfections for 24 h, A549 cells were infected by WNV_KUN_, ZIKV, or LGTV with 0.1 multiplicity of infection. Supernatants were harvested 24 h post-infection and cells were detached by trypsin, followed by a soybean trypsin inhibitor treatment (Gibco). Cells were then briefly frozen in liquid nitrogen and thawed repeatedly three times.

### 2.7. Antibodies

The following antibodies were used in this study: rabbit anti-LNP (Atlas Antibody, Stockholm, Sweden), monoclonal mouse-LGTV E (clone 11H12, United States Army Medical Research, Institute of Infectious Diseases, Fort Detrick, Frederick, MD, USA), mouse anti-mCherry tag (Novus Biologicals, Englewood, CO, USA), rabbit anti-mCherry tag (Novus Biologicals, USA), mouse anti-GAPDH (Sigma, St. Louis, MO, USA), Alexa Fluor 594-conjugated anti-mouse goat antibody (Invitrogen), Alexa Fluor 488-conjugated anti-rabbit goat antibody (Invitrogen), anti-mouse VisUCyte HRP Polymer (R&D Systems, Minneapolis, MN, USA) and HPR-conjugated anti-mouse goat antibody (Invitrogen).

### 2.8. Plaque Assays

Crystal violet-based plaque assays were performed to quantify infectious WNV_KUN_ and ZIKV particles, while immuno-focus plaque assays were performed to quantify infectious LGTV. In brief, series of virus dilutions in DMEM were used to infect 90% confluent Vero cells for 1 h at 37 °C, followed by cell-overlaying with DMEM supplemented with 1.2% Avicel (FMC, Philadelphia, PA, USA), 2% HI‒FBS, 1X NEAA, 1% PEST. After 3–4 days, the overlays were removed, and cells were fixed with methanol for 20 min before performing plaque assays. For immuno-focus assay, the fixed cells were blocked by 2% BSA for 10 min before being labeled with 1:1000 mouse LGTV E, then 1:100 VisUCyte anti-mouse secondary HRP Polymer for 1 h at 37 °C, respectively. Finally, the cells were incubated with KPL TrueBlue Peroxidase Substrate (Seracare, Milford, MA, USA) for 20 min at room temperature (RT). For crystal violet-based plaque assay, the fixed cells were stained with 1% crystal violet (Sigma), 20% methanol (Fisher, Trinidad and Tobago), and 1% ammonium oxalate (Sigma) solution.

### 2.9. Quantitative Real-Time PCR (qPCR)

Total RNA was isolated from cell lysates or cell culture supernatants using RNeasy Mini kit (Qiagen) or QIAamp Viral RNA Mini Kit (Qiagen), respectively. cDNA was synthesized using a High-Capacity cDNA Reverse Transcription kit (Applied Biosystems, Vilnius, Lithuania) with specific primers targeting the WNV_KUN_, ZIKV, or LGTV positive strand (Table 1). qPCR was conducted using a QuantStudio 7 Flex Real-Time PCR System (Applied Biosystems) with TaqMan Fast Advanced Master Mix (Applied Biosystems) and Custom TaqMan Gene Expression Assays (Applied Biosystems) containing specific primers and probes (Table 1).

### 2.10. Luciferase Assay

Cell lysates were assayed for bioluminescence using Dual-Luciferase Reporter Assay kit (Promega, USA) at a Lumi-star Omega (BMG Labtech, Ortenberg, Germany), according to the manufacturer’s instructions.

### 2.11. Protein Electrophoresis and Immunoblotting

Cells were lysed by 0.5% SDS prior to boiling in 1X LDS sample buffer (Invitrogen). Proteins were separated on precast 4–12% polyacrylamide Bis-Tris gels (Invitrogen) in MES running buffer (Invitrogen) for 60 min at 120 V constant before being transferred to nitrocellulose membranes using iBlot 2 Gel Transfer Device (Invitrogen). Proteins of interest were detected with the antibodies anti-mCherry (1:1000), anti-LNP (1:1000), and anti-GAPDH (1:2000).

### 2.12. Immunoprecipitations (IP)

For protein-protein interaction studies, A549 cells were transfected with the gene constructs using a NucleofectorTM 2b Device (Lonza, Basel, Switzerland) and Cell Line NucleofectorTM Kit T (Lonza). Two days post-transfection, the cells were lysed by radioimmunoprecipitation assay (RIPA) buffer (Sigma) supplemented with a protease inhibitor cocktail (Sigma) for 20 min at 4 °C. Lysates were pre-cleared by centrifuge at 1000× *g* for 10 min. 300 μg of the pre-cleared lysates were incubated with 4 μg of mouse anti-mCherry, 50 μL of a protein G-tagged microbead pre-mix (MACS, Stockholm, Sweden) at 4 °C for 1 h. The mixtures were then loaded on μ Columns (MACS) holding on a μMACS Separator to retain the microbeads on the columns. The beads were washed four times with 4 °C 50% RIPA buffer. Finally, bead-binding proteins were eluted by a pre-heated 95 °C LDS sample buffer.

### 2.13. Transmission Electron Microscopy (TEM)

Cells were fixed in 2.5% glutaraldehyde (Ladd, Bureau County, IL, USA) in 0.1 M phosphate buffer pH 7.4 at RT for 1 h, followed by storage at 4 °C. Following the primary fixation, the cells were rinsed with 0.1 M phosphate buffer and post-fixed in 2% osmium tetroxide in 0.1 M phosphate buffer, pH 7.4 at 4 °C for 2 h. The cells were then ethanol dehydrated stepwise, followed by stepwise acetone/LX-112 infiltration, and finally embedded in LX-112 (Ladd). Semi-ultrathin sections were prepared using the EM UC 7 (Leica, Wetzlar, Germany). The ultrathin sections (approximately 60–80 nm) were contrasted with uranyl acetate followed by Reynolds’ lead citrate and examined in an HT7700 transmission electron microscope (Hitachi High-Tech, Tokyo, Japan) operated at 100 kV. Digital images were acquired using a 2k × 2k Veleta CCD camera (Olympus Soft Imaging Solutions GmbH, Münster, Germany).

### 2.14. Immunofluorescence Labeling

A549 cells grown on coverslips were fixed by 4% paraformaldehyde (Scharlau, Barcelona, Spain) for 20 min, followed by permeabilization by 0.1% Triton X-100 (VWR) before blocking with 2% bovine serum albumin (BSA) (Fitzgerald, Crossville, TN, USA) and 2% goat serum (Invitrogen) for 1 h. Cells were labeled with the primary antibody anti-LNP (1:250) for 1 h at 37 °C, followed by incubation with the secondary antibody Alexa Fluor 488 (1:1000) under similar conditions. Images were captured using a confocal laser scanning microscopy SP8 (Leica, Germany) and analyzed using ImageJ.

### 2.15. Statistics

Statistical differences between the means were determined using Student’s *t* test or one-way ANOVA followed by Bonferroni post-test, which *p* < 0.05 was considered to indicate a statistically significant difference in the comparison of groups. GraphPad Prism was used for performing all statistical analyses. The values are presented as mean ± standard error of the mean.

## 3. Results

### 3.1. LNP Is Essential for WNVKUN, ZIKV, and LGTV Production

**Figure 1:**
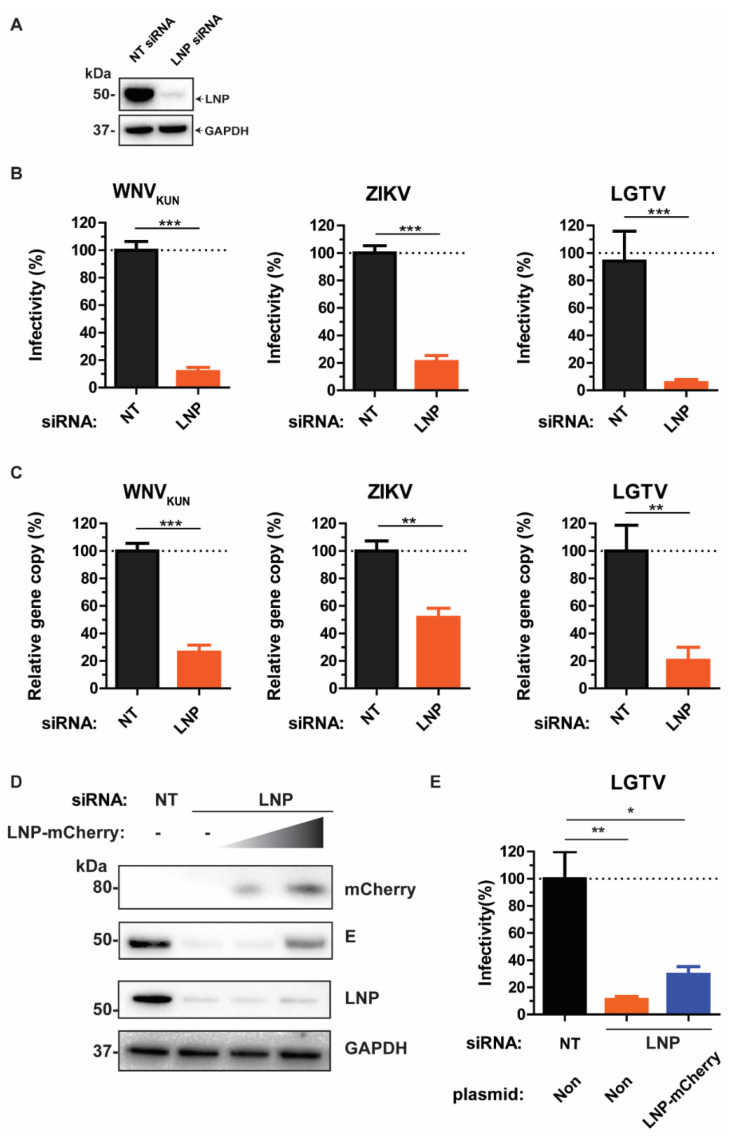
LNP is essential for flavivirus production. (**A**) Immunoblotting of cell lysates 48 h post-transfection with the antibody against LNP after non-targeting (NT) and LNP-targeting siRNA transfection. The expression levels were normalized using the endogenous glyceraldehyde-3-phosphate dehydrogenase (GAPDH) protein as a control. (**B**) The percentages of infectious virus particles released in the supernatants of LNP-silencing cells in comparison with NT-silencing cells after WNV_KUN_, ZIKV, and LGTV virus infections, measured by plaque assays. (**C**) The percentages of gene copy number of viruses released in the supernatants of LNP-silencing cells in comparison with the control, measured by qPCR. (**D**) Immunoblotting of cell lysates after siRNA treatment, transfection with a titer of LNP-mCherry, and infection with 0.1 MOI LGTV. The proteins were visualized with antibodies against mCherry, LNP, LGTV E, and GAPDH as the loading control. (**E**) The percentages of infectious virus particles released in the supernatants of LNP-silencing cells followed by transfection of a high quantity of LNP-mCherry DNA plasmid. The experiments were conducted independently three times with two technical repeats. The *p* values are indicated using * *p* < 0.05, ** *p* < 0.01, and *** *p* < 0.001.

The membrane-remodeling protein, LNP plays a role in maintaining the three-way tubular junctions within the ER [16,17], which has highly curve structures like the Ve. Thus, we sought the effects of the endogenous LNP during flavivirus infection. Initially, LNP depletion in A549 cells was mediated by LNP siRNA transfection. LNP knockdown was demonstrated by immunoblotting of the cell lysates with anti-LNP antibody (Figure 1A).

We infected the LNP knockdown cells and cells transfected with the non-targeting (NT) control siRNA with WNV_KUN_, ZIKV, or LGTV, followed by determination of the virus titers from the cell culture supernatants, respectively. There were 80–90% reductions of virus titers during LNP depletion compared to the NT control (Figure 1B). Consistently, there were reductions in gene copy numbers of these viruses measured by qPCR during LNP depletion (Figure 1C). During ZIKV infection, we observed around 50% of reduction in gene copy number in comparison with the control, whereas 70‒80% of reductions were detected during WNV_KUN_ and LGTV infection, respectively. Collectively, the similar inhibitory trends between virus titers and gene copy numbers suggest the effect of LNP-silencing occurs prior to the virus maturation stage.

To verify the specificity of siRNA silencing, we rescued the virus production phenotype during LNP depletion. As it has been shown that the normal ER structures during LNP removal can be regenerated only by overexpressing certain levels of LNP [23], LNP- depleted cells were transfected with a titer of LNP-mCherry construct, followed by infection with 0.1 MOI of the LGTV. By immunoblotting of cell lysates, there was an increase of the LGTV E protein at high LNP-mCherry-expressing conditions (Figure 1D). Similarly, there was a partial rescue of LGTV production in the supernatant by transfecting cells with a high amount of LNP-mCherry, compared to non-transfection of LNP-mCherry (Figure 1E). Thus, these data suggest a specific role of LNP for LGTV production.

### 3.2. Requirement of LNP for Intracellular WNV_KUN_, ZIKV, and LGTV

**Figure 2:**
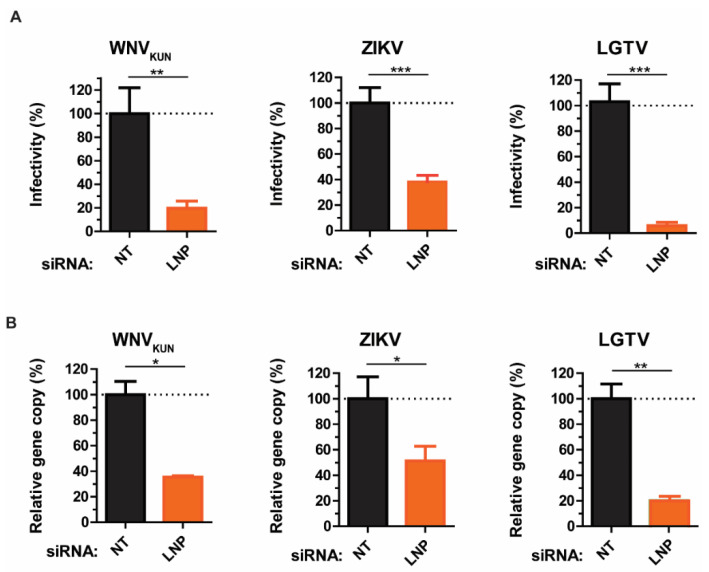
Reductions of intracellular virus titers during LNP depletion. (**A**) The percentages of infectious virus particles in the LNP-silencing cell lysates in comparison with NT controls after WNV_KUN_, ZIKV, and LGTV infections measured by plaque assays. (**B**) The percentages of virus gene copy numbers measured by qPCR from the LNP-silencing cell lysates in comparison with the controls. The experiments were conducted independently three times with two technical repeats. The *p* values are indicated using * *p* < 0.05, ** *p* < 0.01, and *** *p* < 0.001.

As we showed that LNP may act prior to virus maturation, we sought to investigate the effects of LNP depletion at earlier stages of the virus life cycle, such as virus assembly and budding. Here, we compared the intracellular infectious particle numbers versus gene copy numbers. siRNA-treated cells were infected with WNV_KUN_, ZIKV, or LGTV, then harvested and repeatedly frozen/thawed three times in liquid nitrogen to release the intracellular virus particles. In accordance with the virus titers in supernatants, there were significant reductions in titers of infectious intracellular virus particles. Silencing of LNP resulted in 80–90% reductions of virus titers, as measured by plaque assays (Figure 2A). Similarly, virus gene copy numbers from cell lysates were reduced due to LNP depletion. Particularly, LGTV infected cells showed the highest reduction of 80% compared to the negative control. This effect was more moderate in ZIKV and WNV_KUN_ infected cells having reductions by 50–60% (Figure 2B), which indicates an important role of LNP during LGTV infection. Taken together, the similarity between results from plaque assays and qPCR from the cell lysates and supernatants suggests LNP does not act on virus release.

### 3.3. LNP Has a Role in WNV_KUN_ and LGTV Replication

**Figure 3:**
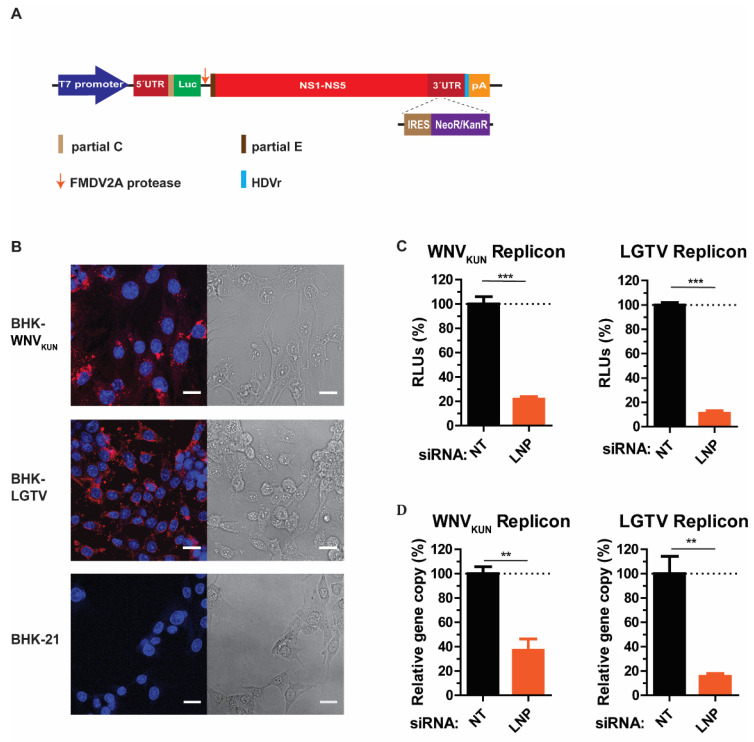
LNP is required for flavivirus replication. (**A**) Schematic illustration of the replicon construct to generate replicon-expressing cell lines. RNA replicons were expressed in vitro by the T7 promotor-driven DNA constructs, which is flanked by the 5′- untranslated region (UTR), the 3′-UTR, and comprises: the firefly luciferase gene (Luc) as a reporter gene in place of genes coding structural proteins, the foot-and-mouth disease virus autoprotease 2a (FMDV 2A), and all the nonstructural proteins of the flavivirus. The antigenomic hepatitis delta virus ribozyme (HDVr) sequence was inserted immediately downstream of the 3′-UTR followed by the simian virus 40 (SV40) polyadenylation signal (pA). An internal ribosome entry site (IRES) sequence and the neomycin/kanamycin resistance (NeoR/KanR) gene were inserted within the 3′-UTR. (**B**) Left panels: Immunofluorescence staining with the dsRNA antibody of the WNV_KUN_ and LGTV replicon-expressing cells. The non-transfected BHK-21 cells were used as the control. The nuclei were counterstained by DAPI (blue). The bar scales represent 20 µm. Right panels: bright field images of the respective cells. (**C**) The percentages of relative luciferase units (RLU) from the BHK-WNV_KUN_ or the BHK-LGTV cell lysates during LNP-silencing in comparison with NT- silencing. (**D**) The percentages of replicon gene copy numbers measured by qPCR from the LNP-silencing cell lysates in comparison with the NT-silencing cell lysates. The experiments were conducted independently three times with two technical repeats. The *p* values are indicated using ** *p* < 0.01, and *** *p* < 0.001.

As our preliminary data suggested that LNP may have an important function during virus replication, we generated cell lines expressing RNA WNV_KUN_ or LGTV replicons, termed BHK-WNV_KUN_ and BHK-LGTV, respectively, to specifically study virus replication. Here, WNV_KUN_ represents a mosquito-borne flavivirus, while the LGTV represents a tick-borne flavivirus. Initially, the RNA replicons were synthesized by in vitro transcription prior to transfection into BHK-21 cells. For replicon synthesis, DNA constructs were created that insert a neomycin/kanamycin resistance (NeoR/KanR) gene, driven by an internal ribosome entry site (IRES), within the 3′-UTR. Resistance to G418 drug killing was conferred to cells expressing the replicon RNA. After selection, the majority of cells expressed the replicating replicons indicated by immunolabeling with dsRNA antibody (Figure 3B). Furthermore, neither BHK-WNV_KUN_ nor BHK-LGTV cells had obvious cytopathic effects compared to the control, as indicated by bright-field images (Figure 3B).

LNP depletion in BHK-WNV_KUN_ and BHK-LGTV cells resulted in significant reductions of replicon expression, monitored by the activity of the luciferase reporter gene (Figure 3C). There was an 80% and 90% reduction of luciferase expression in LNP-depleted BHK-WNV_KUN_ and BHK-LGTV cells, respectively. In accordance with the luciferase expression, the replicon gene copy numbers were also reduced by 60% or 80% due to the LNP silencing in BHK-WNV_KUN_ or BHK-LGTV cells, respectively. Altogether, these results suggest that LNP acts during flavivirus replication.

### 3.4. Function of LNP in Regulating the Number and the Size of Replication Vesicles (Ve)

**Figure 4:**
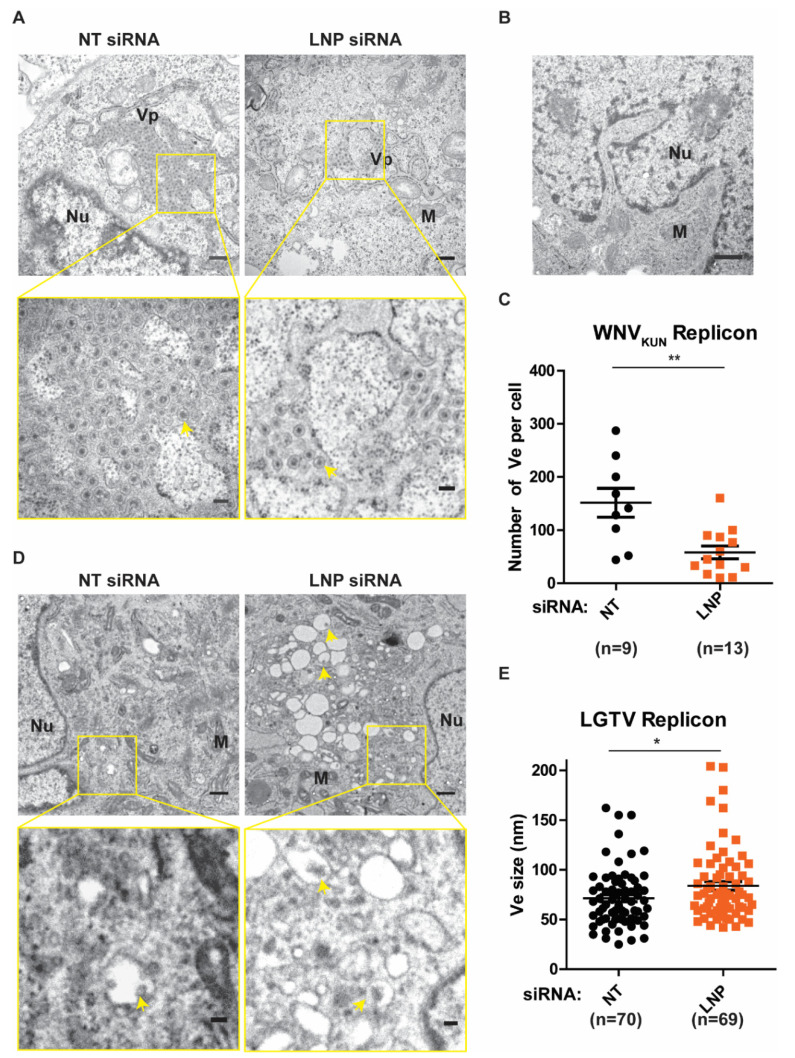
LNP depletion reduced virus-induced vesicles (Ve) in the replicon expressing cell lines. (**A**) Ultrastructural analysis of BHK-WNV_KUN_ cells transfected with NT or LNP siRNA by transmission electron microscopy (TEM). The lower panels show higher magnification images of the yellow boxes indicated in the upper panels, respectively. Scale bars represent 500 nm in the upper panels and 100 nm in the lower panels. Arrows indicate the Ve, ER: the endoplasmic reticulum; Nu: nucleus; M: mitochondria; Vp: vesicle packets. (**B**) TEM image of the BHK-21 cells having no replicons. Scale bar represents 500 nm. (**C**) The number of Ve per WNV_KUN_ cell after LNP deletion compared to the NT silencing control. (**D**) TEM images of BHK-LGTV cells transfected with the indicated siRNA. The lower panels show higher magnification images of the yellow boxes indicated in the upper panels, respectively. Scale bars represent 500 nm in the upper panels and 100 nm in the lower panels. (**E**) The size of Ve in the BHK-LGTV cells. The *p* values are indicated using * *p* < 0.05 and ** *p* < 0.01.

After we have observed the inhibitory effects on flavivirus replication during LNP silencing, we sought to verify the function of LNP on generation and maintenance of the Ve, assuming it to be the underlying mechanism of reductions in replication. Initially, we analyzed these ER membrane ultrastructures in BHK-WNV_KUN_ cells during LNP depletion compared to the control NT-silencing (Figure 4A). In these cells, the ER membrane is remodeled to generate clusters of Ve, similarly to the duration of the virus infection [6,7], but not in the naïve BHK-21 cells (Figure 4B). We evaluated the number of Ve per cell during LNP silencing versus NT silencing. Interestingly, there was a significant reduction in the numbers of Ve in the LNP-depleted cells compared to the control cells (Figure 4C). Similarly, we observed that there were Ve in the BHK-LGTV cells (Figure 4D) as previously described [24,25,26,27]. Interestingly, depletion of LNP resulted in enlargements of the Ve (Figure 4D,E), suggesting defects in the maintenance of the Ve curvature. However, LNP depletion did not alter the number of Ve per cell in the BHK-LGTV cells. Altogether, these results suggest that the reason for reduced replication during LNP depletion is due to the role of LNP in the Ve generation or maintenance.

### 3.5. LNP Interacts with NS4B

**Figure 5:**
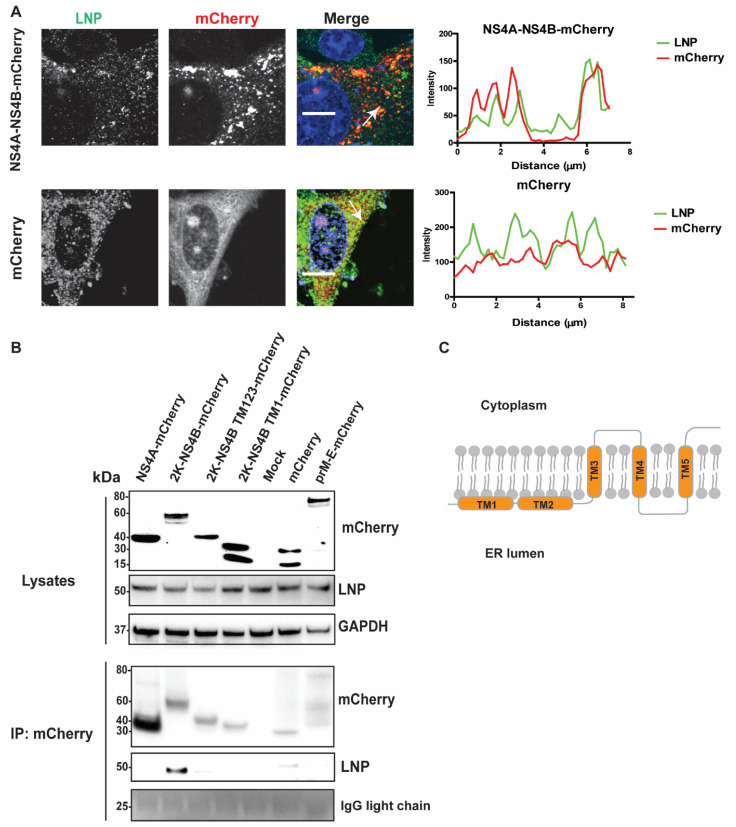
LNP co-localizes and interacts with NS4B. (**A**) Immunofluorescence labeling of cells transfected with NS4A-NS4B-mCherry compared to cells transfected with mCherry. A549 cells were visualized with anti-LNP antibodies (green) and mCherry (red). Nuclei were counterstained with DAPI (blue). Bar scales represent 10 µm. Graphs on the right panel illustrate green and red fluorescent intensity at white arrows in the left images, respectively. (**B**) Immunoblotting of cell lysates 48 h post transfection of the NS4A, 2K-NS4B, 2K-NS4B transmembrane (TM) domain 123, 2K-NS4B TM1, anchored prM-E constructs having the mCherry tag at the protein C-terminal, or the mCherry construct, prior to immunoprecipitation by anti-mCherry beads. The proteins were visualized with antibodies against mCherry, LNP, and GAPDH as the loading control. (**C**) Schema illustrates structure of the polytopic NS4B protein with 5 TM domains spanning the ER membrane.

Since we showed that LNP has a function in viral replication and Ve generation, we sought to determine if the protein specifically interacts with viral proteins of the RC. It has been indicated previously that depletion of LNP resulted in a strong inhibitory effect on LGTV, which is the naturally attenuated virus in the TBEV serocomplex [28,29]. To explore LNP interactions with RC components, we first looked for visual recruitment of LNP to viral proteins. We focused our study on the TBEV Europe type NS4A and NS4B proteins that are known to generate Ve [8,9,10,30]. We expressed a mCherry protein or a mCherry-tagged NS4A-NS4B fusion protein in A549 cells and then immunostained the cells with an anti-LNP antibody (Figure 5A). By measuring the intensity of fluorescent signals representing mCherry versus LNP, we showed that there is close localization between LNP and NS4A-NS4B-mCherry in comparison with the control mCherry (Figure 5A). During the NS4A-NS4B-mCherry expression, we observed fluorescent dots indicative of Ve and the site of LNP recruitment (Figure 5A, upper panel).

As we determined the close localization between NS4A-NS4B-mCherry and LNP at the induced Ve, we sought to investigate if there are direct interactions between these proteins and LNP. We ectopically expressed NS4A, 2K-NS4B, 2K-NS4B TM1–3, 2K-NS4B TM1, or anchored prM-E having the mCherry tag at the protein C-terminus and then immunoprecipitated (IP) mCherry-containing protein complexes from cell lysates with the mCherry antibody (Figure 5B). Here, the anchored prM-E-mCherry acts as a membrane protein control. Furthermore, the 2K domain and the anchored domain of capsid protein in the 2K-NS4B and the anchored prM-E constructs, respectively, allowed the proteins to locate at the ER membrane with correct topologies. During expression, both the 2K and the prM are cleaved from these proteins by host peptidase results in NS4B-mCherry and E-mCherry, respectively [8,31]. LNP co-precipitated from the cell lysates expressing 2K-NS4B-mCherry, but not from NS4A or the other controls (mCherry or prM-E), indicating the interaction between LNP and NS4B (Figure 5B). Interestingly, removal of the TM4 and TM5 of NS4B (Figure 5B,C) aborted the interaction between NS4B and LNP. Overall, the data suggest that LNP is recruited to the RC and that interactions between LNP and the C-terminal region of NS4B is important.

## 4. Discussion

Compared to cellular organisms, viruses have a limited genome. During cellular infection, viruses often employ host cell factors for their life cycle and develop mechanisms to delay the host defense system [32,33]. In this study, we attempted to characterize the functions of LNP during the flavivirus life cycle. Initially, we compared the numbers of infectious viral particles with the viral gene copy numbers from the supernatants to evaluate alterations in the viral maturation stage. Then we compared the intracellular and extracellular viral particles to assess changes in virus release. Interestingly, we observed stronger inhibitions during depletions of LGTV compared to WNV_KUN_ and ZIKV, suggesting the preference utility of LNP by tick-borne flaviviruses.

In addition, we generated cell lines expressing the WNV_KUN_ or LGTV replicons, which represent mosquito-borne and tick-borne flaviviruses, respectively, to specifically study viral replication. Our replicon platforms did not show major cytopathic effects and maintained the replicon expression for many passages (data not shown), which is similar to a previously described system [34]. We showed that insertion of the IRES-NeoR/KanR in a variant region of the 3′UTR did not abolish the replication capacity of the replicons but conferred an antibiotic resistance capacity to transfected cells. These cell lines can be good systems to study proteins of the host machinery having roles in the virus replication such as LNP in our study and can be platforms for screening antiviral-compounds targeting the virus replication.

In this study, we showed that LNP depletion resulted in defective replication of WNV_KUN_ and LGTV. These inhibitory effects were due to the role of LNP in either induction or stabilization of the Ve. Interestingly, the protein interacts specifically with NS4B that is a TM protein scaffolding the RC. The protein is linked to NS4A by the 2K domain that is initially cleaved by NS2B/NS3 to form 2K-NS4B [8], followed by the host signal peptidase SPCS1-mediated cleavage to remove the 2K [8,35]. NS4B is a conserved protein in flaviviruses with five integral TM domains (Figure 5C), mutations of which abrogate virus replication and decrease virulence [36]. The protein contains multiple interaction motifs for dimerization, interaction with other NS proteins in the RC and host proteins [36]. In our study, we found that the C-terminal region of NS4B containing the TM4‒5 may be essential for the interaction of the protein with LNP.

In eukaryotic cells, the ER is a dynamic constant-remodeling membranous organelle spreading throughout the cytoplasm [37]. This network consists of sheets and tubules interconnected by three-way junctions. The conserved membrane protein LNP localizes at the ER junctions and stabilizes them [17], as depletions of the protein have resulted in a densely sheet-like ER [16,23,38]. The ER phenotype can be rescued by overexpression of LNP at a certain level [23]. In our study, we partially rescued the LGTV production by overexpressing LNP from the LNP-mCherry construct. We speculate that higher virus production can be rescued by using other LNP construct having codons optimized to resist siRNA or by higher overexpression of LNP construct in cell lines stably depleting LNP.

Furthermore, the LNP protein has been shown to be ubiquitinated and recruit the rapamycin complex-1 at the ER three-way junction and lysosomes. Inhibition of LNP ubiquitination triggers neurodevelopmental defects [39]. In this study, we propose a role of LNP during flavivirus replication as the protein is recruited to the Ve, mainly located at the ER [40], by its interaction with NS4B. However, the question that the LNP‒NS4B interaction can hinder the LNP ubiquitination, resulting in the neural cytopathic effects during flavivirus infections, remains for future studies.

Other ER three-way junctions inducers and stabilizers have also been characterized during flavivirus infection including RTN [41,42], DP1 [12,13], and ATL [14,15]. Indeed, RTN3 has been shown to act in the formation of the Ve for WNV_KUN_, Dengue virus serotype 2 (DENV-2), and ZIKV, by interacting with NS4A [18]. In that study, depletions of RTN3 significantly inhibited the intracellular gene copy number of ZIKV but was less effective with DENV-2 and WNV_KUN_ [18]. In addition to flaviviruses, this protein is also employed for the replication of other RNA viruses, including enterovirus A71 [43], brome mosaic virus, and hepatitis C virus [44]. Furthermore, silencing of SEC61β that interacts with RTN1 [45] resulted in a reduction of WNV_KUN_ and DENV-2 production [35]. In addition to the RTN family, the endogenous ATL2 [19] has been shown to function in replication and formation of the Ve in DENV-2 and ZIKV but not WNV (strain NY99) [19] by its interaction with NS3. Contradictory, LNP and RTN3 appeared to have no effect on DENV-2 from the study [19]. In our study, we have shown that LNP plays a role in Ve formation and replication for two neurotropic flaviviruses, WNV_KUN_ and LGTV. We also demonstrated that the protein interacts with NS4B and that TM4–5 may have a direct role in the interaction. Therefore, these observations indicate the divergence of flaviviruses in employing the orchestra of ER-shaping proteins: LNP, RTN, and ATL for Ve generation. Interestingly, LNP has been demonstrated to act in synergy with RTN but antagonism to ATL to remodel the ER [16,38], which suggests enhanced anti-flavivirus conditions for future investigations.

## 5. Conclusions

In conclusion, this study has contributed new understandings of the role of host proteins during flavivirus replication. Our results suggested that LNP is important for the Ve formation during the replication of the mosquito-borne WNV_KUN_ and the tick-borne LGTV using the established replicon-expressing cell lines. LNP has also been shown to interact with the C-terminus of NS4B.

## Figures and Tables

**Table 1:** List of primers and probes used for qPCR.

Assay	Virus	Sequence (5′–3′)
cDNA synthesis	WNV_KUN_	AATATGCTGTGTTGTTGTGG
	ZIKV	GATCTTGGTGAATGTGAACG
	LGTV	CTCCCTGTGAGTTCATAATTGG
qPCR assay	WNV_KUN_	CAGACCACGCCATGGCG
		CTAGGGCCGCGTGGG
		FAM-TCTGCGGAGAGTGCAGTCTGCGA-NFQ
	ZIKV	CCGCTGCCCAACACAAG
		CCACTAACGTTCTTTTGCAGACAT
		FAM-AGCCTACCTTGACAAGCAGTCAGACACTCAA-NFQ
	LGTV	ACTGAACTGGAGAAGGAGGA
		CCACAGTCCCATGACGATAAG
		FAM-TAGGCTTGATTGCCTCGGCCTTTC-NFQ

## Data Availability

The data presented in this study is available in this article. Remaining data supporting reported results is available from the corresponding authors upon reasonable requests.
